# Development of pink-beam 4D phase CT for *in-situ* observation of polymers under infrared laser irradiation

**DOI:** 10.1038/s41598-019-43589-6

**Published:** 2019-05-22

**Authors:** Karol Vegso, Yanlin Wu, Hidekazu Takano, Masato Hoshino, Atsushi Momose

**Affiliations:** 1 JASRI, 1-1-1 Kouto, Sayo-cho, Sayo-gun, Hyogo, 679-5198 Japan; 2 Institute of Multidisciplinary Research for Advanced Materials, Tohoku University, 2-1-1 Katahira, Aoba-ku, Sendai, Miyagi 980-8577 Japan

**Keywords:** Imaging and sensing, X-rays

## Abstract

Four-dimensional phase computed tomography (4D phase CT) by an X-ray Talbot interferometer (XTI) with white synchrotron radiation has ever been demonstrated at a temporal resolution of about 1 s for soft-matter samples. However, the radiation damage to samples caused by white synchrotron radiation occasionally hampers our understanding of the sample dynamical properties. Based on the fact that XTI functions with X-rays of a bandwidth up to ca. 10% with performance comparable to that by monochromatic X-rays, filtering white synchrotron radiation to generate a ‘pink-beam’ of a 10% bandwidth is effective to reduce radiation damage without degrading the image quality and temporal resolution. We have therefore developed pink-beam 4D phase CT at SPring-8, Japan by installing a multilayer mirror with a 10% bandwidth and a 25 keV central photon energy. XTI optimal at this photon energy was built downstream, and a CMOS-based X-ray detector was used to achieve fast image acquisitions with an exposure time of 1 ms (or 0.5 ms) per moiré image. The resultant temporal resolution of pink-beam 4D phase CT was 2 s (1 s). We applied the pink-beam 4D phase CT to *in-situ* observation of polypropylene, poly(methyl methacrylate), and polycarbonate under infrared laser irradiation (1064 nm). The dynamics of melting, bubbling, and ashing were successfully visualized in 3D movies without problematic radiation damage by synchrotron radiation.

## Introduction

Computed tomography (CT) is an excellent non-destructive technique that maps X-ray absorption (attenuation) coefficient three-dimensionally in the case of X-ray absorption CT^1^ or X-ray refractive index in the case of X-ray phase CT^2^. X-ray phase CT has outstanding applicability to 3D visualization of the inner structure of weakly absorbing (soft) materials, and the development of X-ray phase CT is mainly aimed at biology, medicine and material sciences such as polymer sciences^3–5^. Recently, X-ray phase CT with XTI^2,6,7^ has been attracting attention because of its flexibility in implementation. One attractive feature is that XTI can be operated with polychromatic X-rays. Therefore, X-ray phase CT with white synchrotron radiation was performed for *in vivo* dynamical observations of living biological specimens^8^, and the changes in inner structure of a living worm, which was considered to be respiratory tract, were visualized with a temporal resolution of 1 s. However, the radiation damage to the worm was serious, resulting in death after just one measurement. Consequently, it was difficult to discuss whether the dynamics observed in the worm were natural or radiation induced. The performance of XTI with X-rays of a bandwidth up to 10% is comparable to that with monochromatic X-rays. However, the bandwidth of white synchrotron radiation from a bending section of a synchrotron radiation storage ring is much broader than that, and the experiment in Ref.^8^ was not optimized for XTI and harmful for samples.

The first action made in this study was to install a band-pass filtering multilayer mirror at BL28B2, SPring-8, Japan, where white synchrotron radiation from a bending section is available for various user experiments. A depth-graded multilayer mirror was designed and fabricated for this purpose, and a pink beam of a 10% bandwidth at a central photon energy of 25 keV and a beam section of 50 mm wide and 5 mm high was generated.

**Figure 1:**
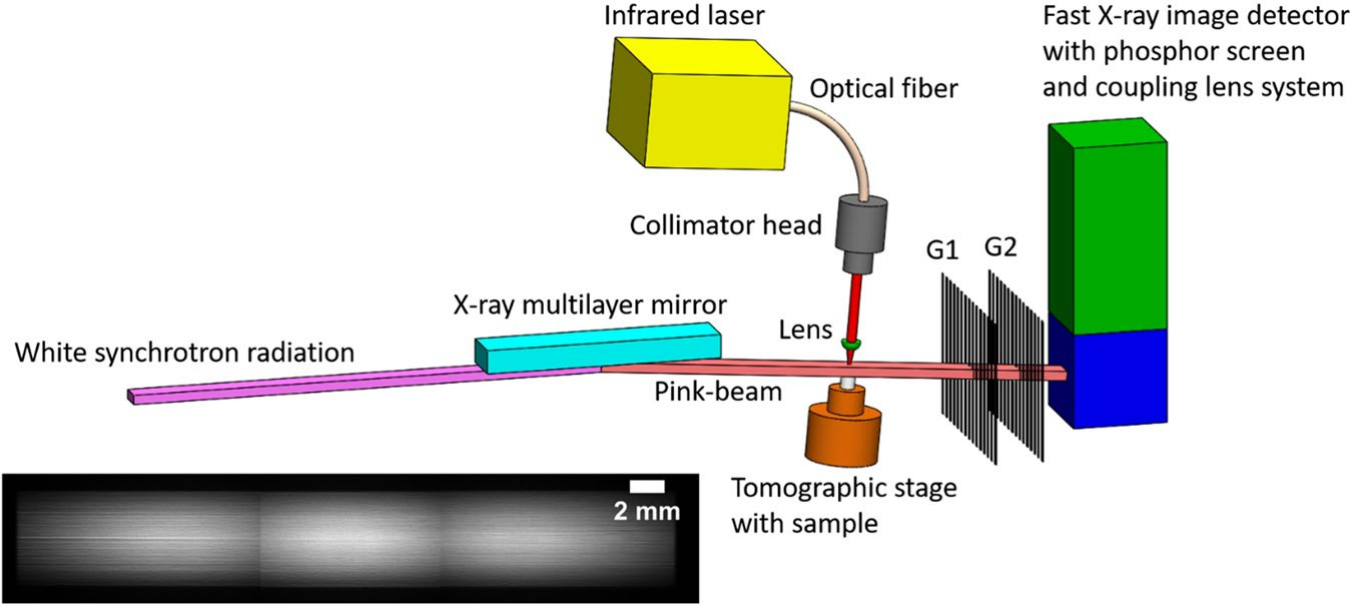
A scheme of a pink-beam 4D phase CT set-up with a multilayer mirror, XTI (G1 and G2), fast X-ray image detector, and an infrared laser source. The image of the pink beam generated from the X-ray multilayer mirror in the inset (the entire beam section (50 mm × 5 mm) is shown by combining three images).

Secondly, we constructed an XTI downstream in combination with a high-speed X-ray image detector, which is composed of a phosphor screen (P46, Hamamatsu Photonics K. K., Japan) and a Complementary Metal Oxide Semiconductor (CMOS) imaging detector via a coupling lens^9,10^. Then, we performed pink-beam 4D phase CT measurements of polymer samples under infrared laser irradiation along the sample rotation axis for CT scan, as shown in Fig. 1. While a hard-X-ray Shack-Hartmann sensor was used for visualizing the laser ablation process in a projection mode^11^, our system depicted melting, bubbling, and/or ashing induced by the laser in movies of 3D phase tomograms with a temporal resolution of 1 s or 0.5 s. We expect that valuable information would be obtained for the field of laser machining (ablation, drilling, cutting, trimming, welding, and so on), which could be used to design the appropriate laser ablation procedures for maintaining polymer strength^12–14^.

## Methods and Materials

### Pink-beam generation

Pink-beam 4D phase CT experiments were performed at BL28B2, SPring-8, Japan where white synchrotron radiation from a bending section is available. A pink-beam was generated by a depth-graded W/Si multilayer mirror (Rigaku Innovative Technologies, Inc., USA), which functions as a band-pass filter against white synchrotron radiation impinging at a grazing angle of 5.11 mrad and generates a 10%-bandwidth X-rays with a 25-keV central photon energy, suppressing the second-order diffraction. The multilayer was formed on a flat Si substrate 50 mm in thickness, 60 mm in width, and 1000 mm in length, and a pink beam (50 mm wide and 5 mm high) was generated by a single bounce downward.

An image showing a reflected beam from the multilayer mirror is shown in the inset of Fig. 1, which is formed by three images because the field of view of the X-ray image detector was smaller than the beam section. The photon flux density downstream of the X-ray multilayer mirror was 10^13^ photons/s/mm^2^. Although weak horizontal stripes caused by the slope errors in the multilayer mirror was seen, this was not crucial as the XTI set downstream was constructed by aligning gratings so that the grating lines were vertical. The spatial coherency in the horizontal direction is sufficiently high at SPring-8, and that in the vertical direction does not matter even if it was degraded by the multilayer mirror to some extent.

### X-ray Talbot interferometer (XTI)

An XTI composed of a π/2 linear phase grating (G1) for 25 keV X-rays and an amplitude linear grating (G2) with an identical pitch of 5.3 μm was built about 8 m downstream of the multilayer mirror and in front of the X-ray image detector (Fig. 1). The distance between G1 and G2 was set to 283 mm, which corresponded to the fundamental fractional Talbot order of a π/2 phase grating at 25 keV. The sample rotation axis was almost vertical (strictly, slightly inclined so that the axis is perpendicular to the pink-beam). Therefore, grating lines were set vertically, and X-ray refraction and scattering in the horizontal direction were measured. The horizontal spatial coherence length, which determined the performance of the XTI, was sufficiently larger than the pitch of the gratings, resulting in 48% visibility of moiré images. A sample was located 150 mm in front of G1.

### Polymer samples

Polypropylene (PP), poly(methyl methacrylate) (PMMA), and polycarbonate (PC) were used in this study. Samples were cut in the form of thick disks, whose diameters (thickness) were 9.8 mm (2 mm), 8 mm (3 mm), and 5 mm (2 mm), respectively. The samples were fixed on an Al holder for CT scan.

The melting temperature and decomposition temperature of PP (PMMA) were 160 °C (160 °C) and 336 °C (226 °C), respectively. The other thermal properties of PP, PMMA and PC are listed in Refs.^15,16^. The average values of the real part δ of the refractive indices decrement from unity for pristine PP, PMMA and PC at a 25-keV X-ray photon energy were 3.41 × 10^−7^, 4.26 × 10^−7^ and 4.2 × 10^−7^, respectively.

### X-ray image detector

A high-speed X-ray image detector was composed of a P46 phosphor screen (YAG:Ce^+^, Hamamatsu Photonics K. K., Japan) 20 μm in thickness and a CMOS imaging detector (FASTCAM SA2, Photron) with a coupling lens system (AA40, Hamamatsu Photonics K. K., Japan)^9,10^. The field of view of the X-ray detector was 15.36 mm (H) × 5.12 mm (V). The effective pixel size of the X-ray detector was 10.1 μm. The X-ray imaging detector was operated at the frame rate of 1000 fps for PP, 2000 fps for PMMA, and 4000 fps for PC. The image size was 1536 (H) × 512 (V) pixels for PP, 1024 (H) × 512 (V) pixels for PMMA, and 768 (H) × 512 (V) pixels for PC. The resultant spatial resolution in combination with the XTI was about 60 μm, which was estimated by evaluating the resultant 4D phase CT images.

### Laser

An infrared Nd:YAG fiber laser (G4, SPI Lasers) of a wavelength of 1064 nm was employed to irradiate samples along their rotation axis via plano-convex lens with a focus size of 35 μm in diameter (FWHM). Continuous-wave (CW) mode and pulse mode were switchable between each other in laser operation. The maximum power of the laser was 50 W for both modes. The laser fluence on the entrance sample surface was 5.2 × 10^6^ J/s/cm^2^ in CW mode.

### 4D phase CT

Phase CT based on XTI reconstructs tomograms that map the distribution of the refractive index from differential phase images, which are conventionally measured by the fringe scanning method (or phase-stepping technique) that moves one of the gratings step by step and acquires a moiré image at every step. Sophisticated sequenceis for grating stepping with a continuous rotation of a sample were reported for phase tomography^17,18^. However, such a procedure involving motions, which displace a grating forward and return it to an origin repeatedly, is not suitable for 4D phase CT, and therefore the continuous fringe scanning (CFS) technique^19^ was used. CFS records the movie of moiré images by moving one of the gratings (in this study, G2) unidirectionally and rotating a sample simultaneously at constant speeds. The speed of the sample rotation was tuned so that the grating displacement was equal to one pitch during five sample rotations. By picking up the frames at the same angular position of sample rotation, the moiré images needed for the calculation of the fringe scanning method are collected.

The sample rotation speeds were 450°/s for PP, 900°/s for PMMA and 1800°/s for PC, and measurements were performed for 56 s, 40 s, and 29 s, respectively. The number of differential phase images obtained per 180° sample rotation was 400 for all CT measurements.

Assuming parallel beam illumination, the data for 180° rotation are used for phase CT reconstruction. Hence, the resultant temporal resolution was 2 s for PP, 1 s for PMMA, and 0.5 s for PC. A series of phase tomograms were reconstructed from the sinogram data in the range from Ω_0_ to Ω_0_ + 180°, increasing Ω_0_ by 45°. Thus, eight tomograms are reconstructed per turn, and the sinogram data for 135° sample rotation were used for neighboring reconstructions for a smooth view of the resultant 4D CT movies.

## Results

### Observation of polypropylene melting

**Figure 2:**
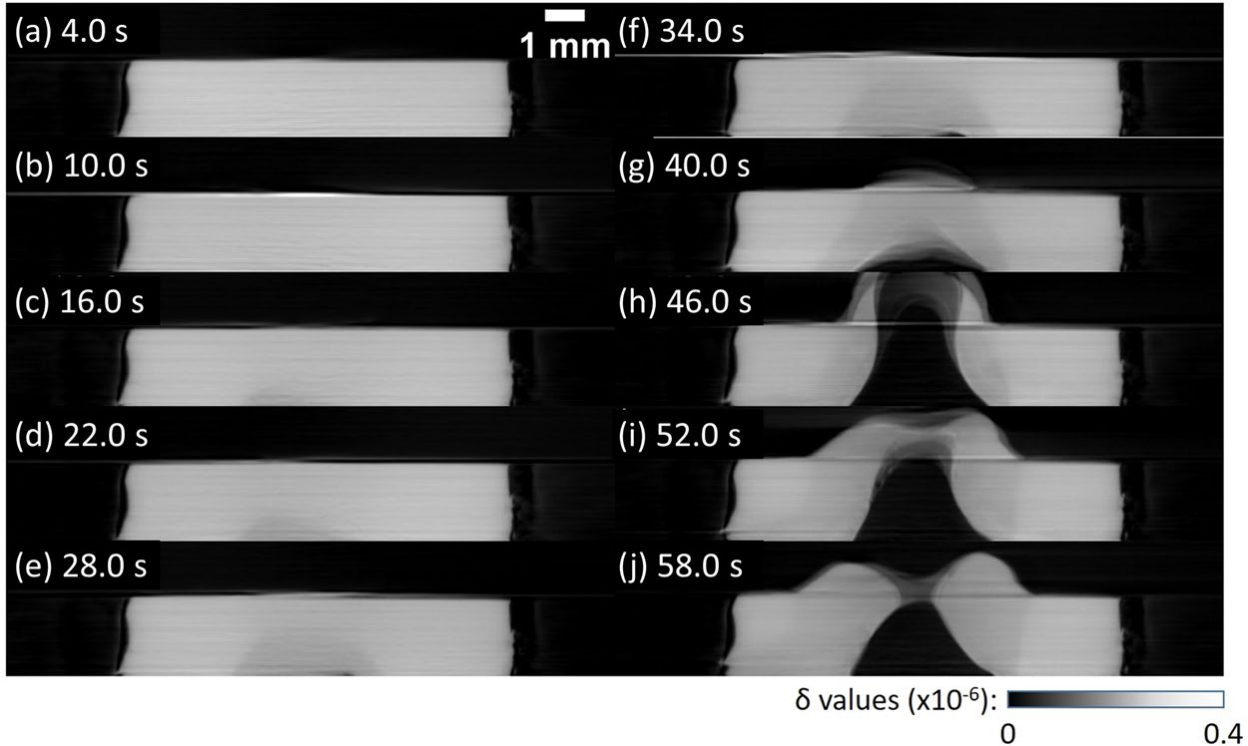
Sagittal section views of 4D phase tomograms obtained for the polypropylene at different laser irradiation times in CW mode: (**a**) 4 s, (**b**) 10 s, (**c**) 16 s, (**d**) 22 s, (**e**) 28 s, (**f**) 34 s, (**g**) 40 s, (**h**) 46 s, (**i**) 52 s, and (**j**) 58 s.

**Figure 3:**
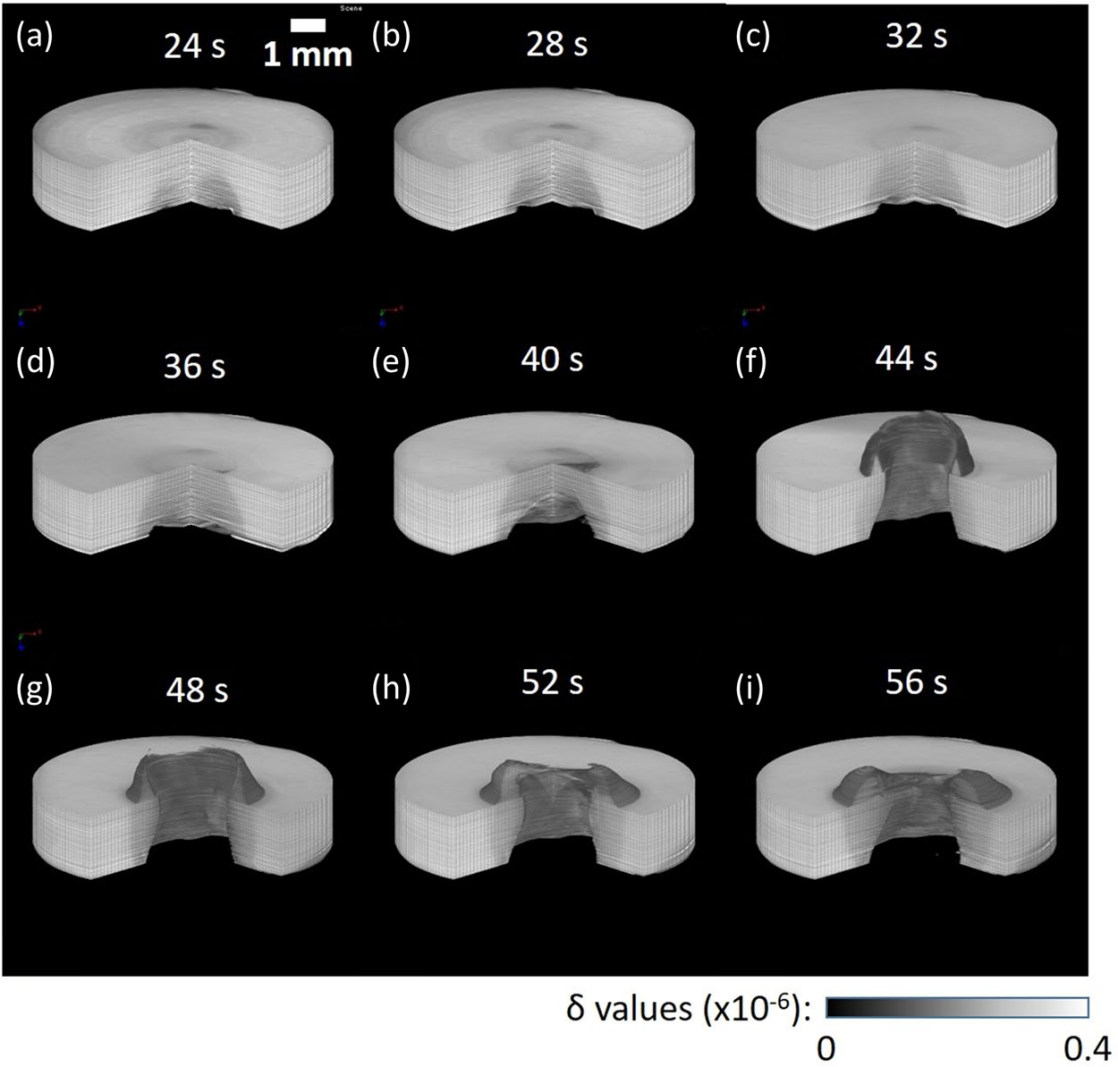
3D rendering views of the 4D phase tomograms obtained for the polypropylene sample at different laser irradiation times: (**a**) 24 s, (**b**) 28 s, (**c**) 32 s, (**d**) 36 s, (**e**) 40 s, (**f**) 44 s, (**g**) 48 s, (**h**) 52 s, and (**i**) 56 s. The morphological changes formed above the entrance sample surface are clearly visible.

Figure 2 shows sagittal slice views on the sample rotation center of the phase tomograms obtained for the PP sample (see also Supplementary Movie S3). The laser operated in CW mode at 50 W was irradiated from the upper surface. As shown in Fig. 2, melting occurred and bubbling (evaporation) followed from the opposite surface, which contacted with the Al holder and was just below the field of view of Fig. 2. The morphological change is shown by volume rendering views in Fig. 3. The optical penetration length of PP at the laser wavelength of 1064 nm is 12.87 mm^14^. According to the Beer-Lambert law, the transmittance of 2 mm thick PP is 85%. Hence, the laser easily penetrates through the PP sample and reaches the Al holder. It is speculated that the heat generated at the Al holder propagated back to the sample.

**Figure 4:**
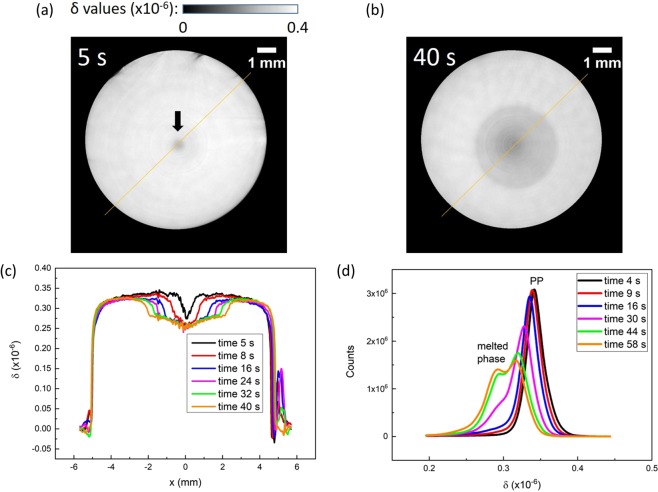
CT slices taken 250 μm below the entrance surface of the PP sample: (**a**) 5 s and (**b**) 40 s of laser irradiation. The arrow in (**a**) shows a spot of melted PP. (**c**) Profiles on the yellow line in (**a**) at different times after starting laser irradiation, showing expansion of the melted region. (**d**) Histograms of δ values at different times after laser irradiation.

The axial slice view at the position 250 μm from the entrance surface at 5 s and 40 s after laser irradiation is shown in Fig. 4(a,b), and the profiles along the yellow line are shown in Fig. 4(c). The δ value of melted PP was within 2.6 × 10^−7^ ~ 2.8 × 10^−7^, while the theoretical δ value of pristine PP is 3.41 × 10^−7^ at 25 keV, which coincides with this measurement. Thus, the boundary of the melted region was differentiated clearly, and the speed of the boundary expansion was evaluated to be 77.9 ± 1.9 μm/s. The histograms of δ values representing tomograms at different times after starting laser irradiation are shown in Fig. 4(d), where a peak emerging at 2.8 × 10^−7^ and corresponding to the melted phase is clearly observable. The regions of melted PP were identified and marked in each CT slice during the segmentation process. The 3D distribution of melted PP within sample at the end of laser irradiation was depicted in Supplementary Fig. S1.

### Observation of bubbling in poly(methyl methacrylate)

**Figure 5:**
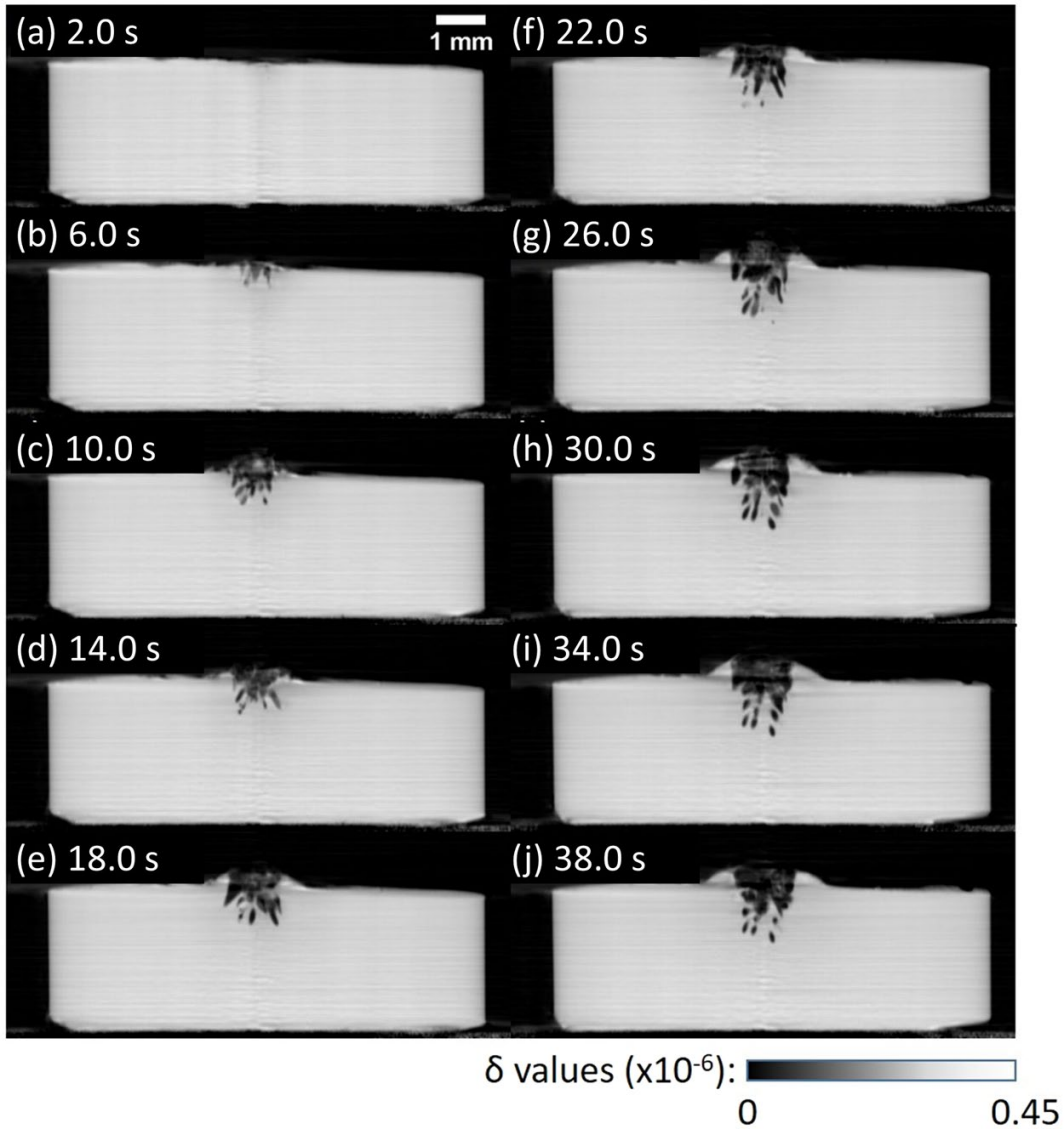
Sagittal section views of 4D phase tomograms obtained for the PMMA sample at different laser irradiation times in pulse mode: (**a**) 2 s, (**b**) 6 s, (**c**) 10 s, (**d**) 14 s, (**e**) 18 s, (**f**) 22 s, (**g**) 26 s, (**h**) 30 s, (**i**) 34 s, and (**j**) 38 s.

**Figure 6:**
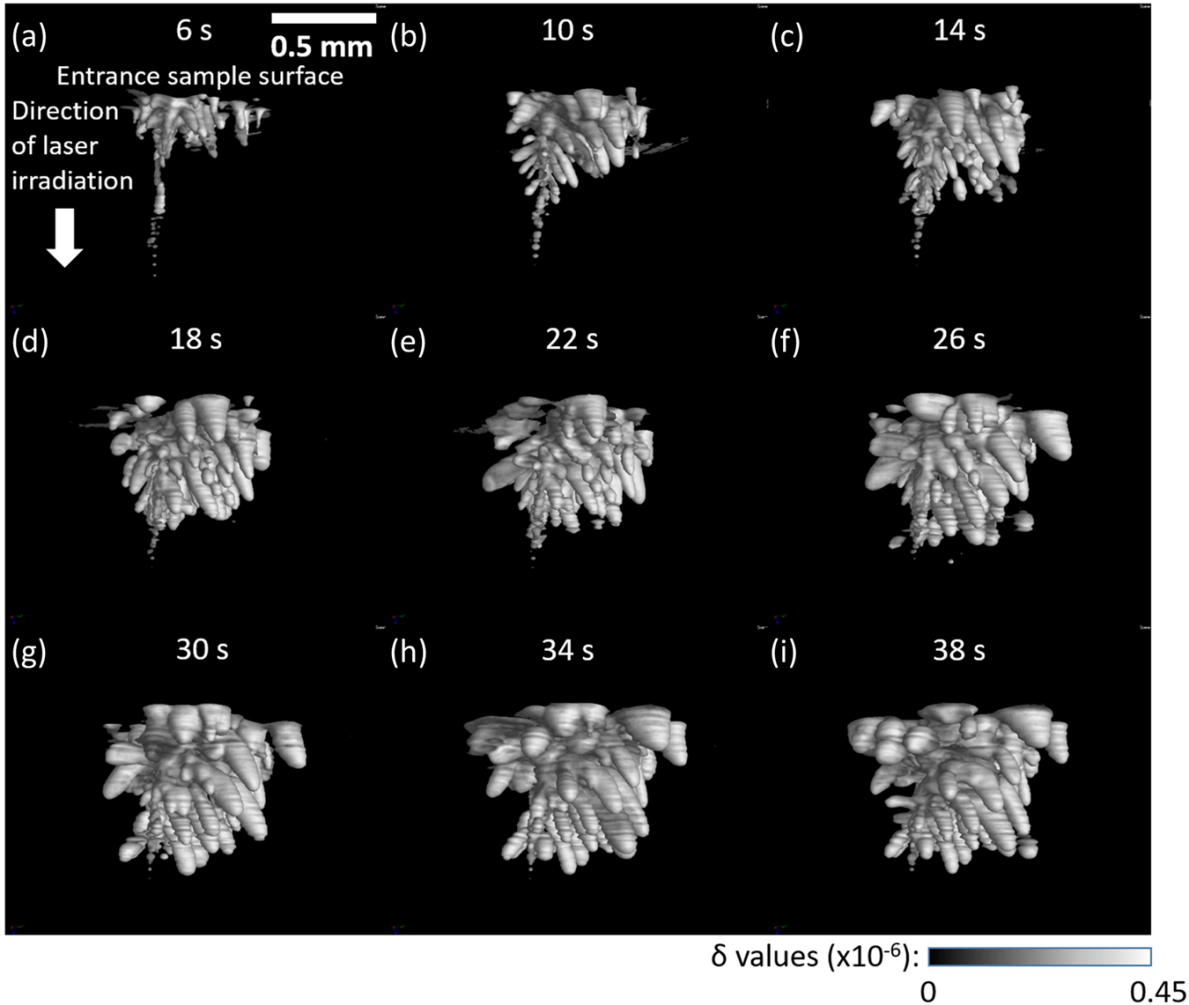
Rendering views of bubbles appeared immediately below laser entrance surface in the PMMA sample at different laser irradiation times: (**a**) 6 s, (**b**) 10 s, (**c**) 14 s, (**d**) 18 s, (**e**) 22 s, (**f**) 26 s, (**g**) 30 s, (**h**) 34 s, and (**i**) 38 s.

The result obtained for the PMMA sample is shown in Fig. 5 (see also Supplementary Movie S4). The laser operated in the pulse mode (pulse width: 50 ns, pulse frequency: 154 kHz) at a peak power of 12 kW and an average power of 50 W. The optical penetration length of PMMA at a wavelength of 1064 nm is approximately 24 mm, which implies that the transmittance for 3 mm thick PMMA is around 88%. However, no change from the Al rod side was observed, unlike the result shown in Fig. 3 and the result in CW mode (see Supplementary Fig. S2). Sagittal section views on the center in Fig. 5 reveal significant structural change (bubbling) at the region hit by the laser. The small bubbles coalesce into larger voids and a hole was made in the PMMA, which is shown in the surface rendering views presented in Fig. 6.

### Observation of polycarbonate ashing

**Figure 7:**
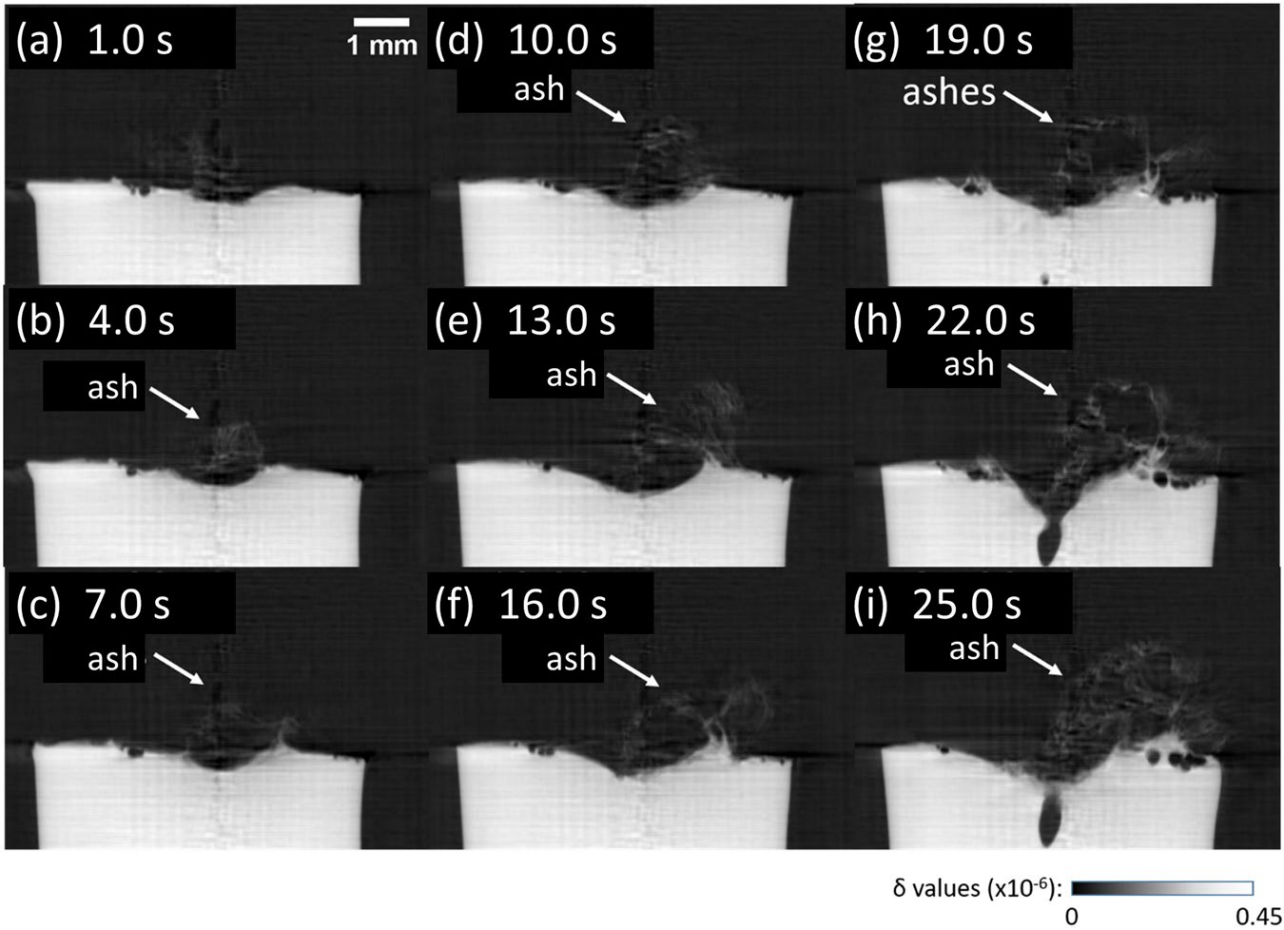
Sagittal section views of 4D phase tomograms obtained for the PC sample at different laser irradiation times in CW mode: (**a**) 1 s, (**b**) 4 s, (**c**) 7 s, (**d**) 10 s, (**e**) 13 s, (**f**) 16 s, (**g**) 19 s, (**h**) 22 s, and (**i**) 25 s. The images show the accumulation of carbon ash on the entrance sample surface.

**Figure 8:**
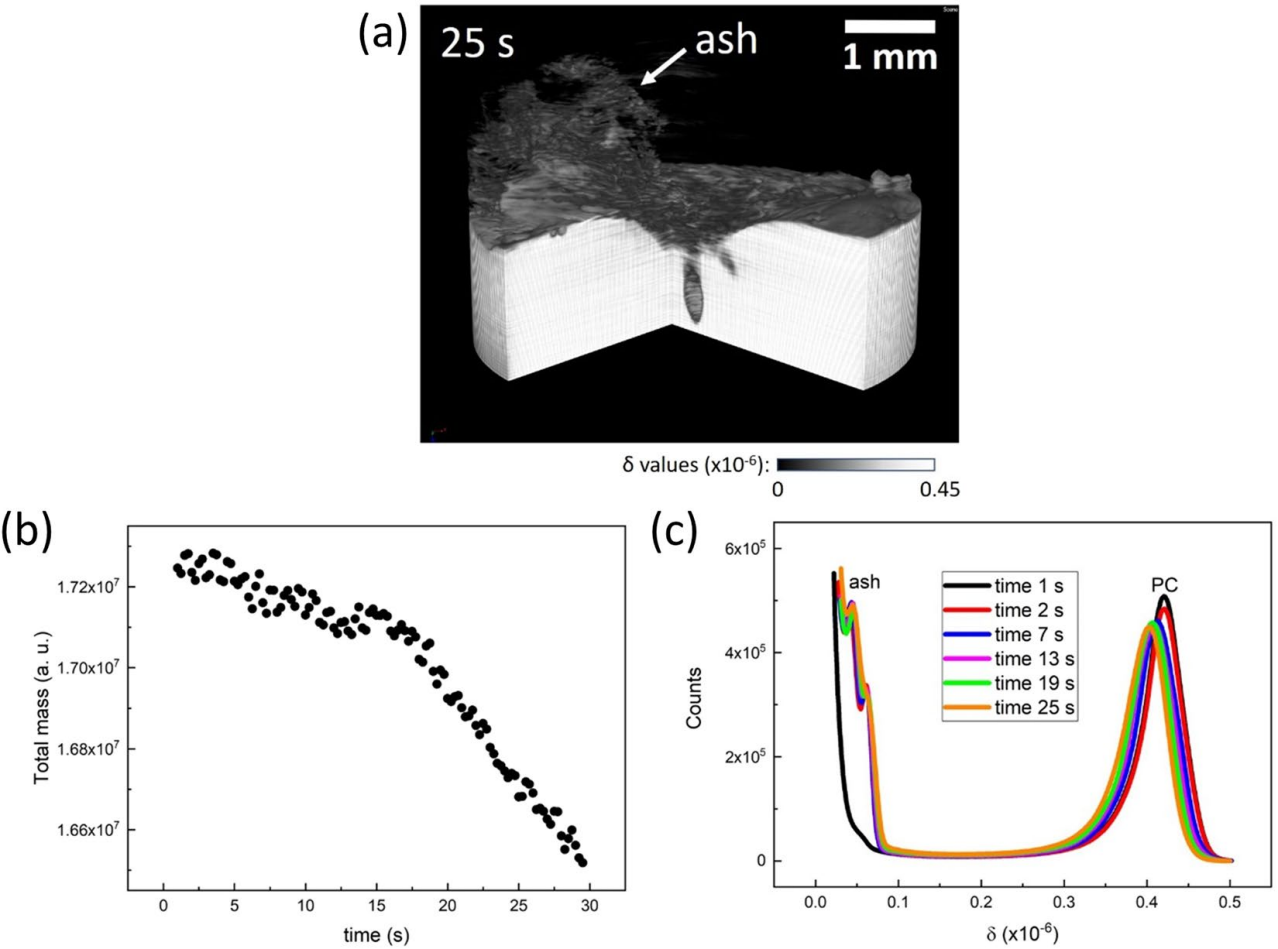
(**a**) 3D rendering view of a phase tomogram obtained for the PC sample at 25 s of laser irradiation. The carbon ash accumulated on the entrance sample surface is depicted. (**b**) The temporal evolution of the total mass of the PC sample during laser irradiation. (**c**) The histograms of δ values calculated for all phase tomograms obtained for the PC sample at different laser irradiation times.

The result obtained for a PC sample in CW mode at 6.55 W is shown in Fig. 7, where carbon ashes were formed (see also Supplementary Movie S5). The 3D reconstruction of the PC sample at 25 s of laser irradiation is depicted in Fig. 8(a), where the formation of carbon ash was successfully visualized. Assuming that the total sum of δ values of all voxels provides total mass, the reduction in the total mass in time is plotted in Fig. 8(b). At the beginning of laser irradiation, ashing was dominated until 17 s. Beyond 17 s, bubbling phenomenon occurred additionally and the total mass decreased more rapidly. The histograms of δ values representing tomograms at different laser irradiation times are seen in the Fig. 8(c), where two peaks corresponding to the pristine PC (δ = 4.2 × 10^−7^) and carbon ash (δ = 0.45 × 10^−7^) can be clearly distinguished.

## Discussion

According to laser ablation theory^16,20^, laser ablation can follow either a “volume absorption” or a “surface absorption” regime. Volume absorption is characterized as the case where the optical penetration length of the polymer is much larger than its thermal diffusion length while it is vice versa in the case of surface absorption. The expansion of melted regions during laser irradiation in PP over several mm (Fig. 2(a)–(i)) suggests “volume absorption” of the thermal energy generated by the laser beam. On the other hand, the interaction of nanosecond pulses with PMMA is characterized by “surface absorption”; that is, the laser was mainly absorbed at the entrance sample surface. Thus, the both regimes of the interaction of laser radiation with polymer materials were demonstrated by pink-beam 4D phase CT.

It is speculated that the result shown in Fig. 2 involved the heat effect from the Al holder in addition to direct interaction of the laser and sample. In order to visualize only polymer laser ablation by 4D phase CT, the contact of the sample with the holder should be avoided by modifying the sample holder (e.g. by drilling a vertical central hole into it).

## Conclusions

A pink-beam of a 10% bandwidth has been generated by a multilayer mirror at BL28B2, SPring-8, and an XTI was combined to perform 4D phase CT. The interaction of infrared laser radiation with PP, PMMA and PC was studied at a spatial resolution of 60 μm and a temporal resolution of 0.5–2 s without causing problematic radiation damage to the samples by synchrotron radiation. Laser-induced PP melting, laser-induced drilling into the PMMA and laser-induced combustion of PC were successfully visualized.

In the near future, we plan to improve the temporal resolution from the current 0.5 s to 80 ms by increasing the frame rate of the X-ray detector from 4000 fps to 10,000 fps. Moreover, the application of pink-beam 4D phase CT will be expanded to laser ablation of polymer composites such as carbon-fiber reinforced polymers, for which scattering contrast generated by fibrous microstructure would be used^21,22^.

## Supplementary information

Supplementary information

Movie S3

Movie S4

Movie S5
