# Supplementary material for: Development of pink-beam 4D phase CT for in-situ observation of polymers under infrared laser irradiation
Source: Sci Rep. 2019 May 22;9:7404. doi: 10.1038/s41598-019-43589-6 (PMC6531456; doi:10.1038/s41598-019-43589-6)
Supplement: Supplementary file 1 — Supplementary information [file 41598_2019_43589_MOESM1_ESM.pdf]

Supplementary information

Development of pink-beam 4D phase CT for *in-situ* observation of polymers under infrared laser irradiation

Karol Vegso[1], Yanlin Wu[2], Hidekazu Takano[2], Masato Hoshino[1] & Atsushi Momose[1,2]

[1]JASRI, 1-1-1, Kouto, Sayo-cho, Sayo-gun, Hyogo 679-5198, JAPAN

[2]Institute of Multidisciplinary Research for Advanced Materials, Tohoku University, Katahira 2-1-1, Aoba-ku, Sendai, Miyagi 980-8577, JAPAN

Corresponding author: atsushi.momose.c2@tohoku.ac.jp

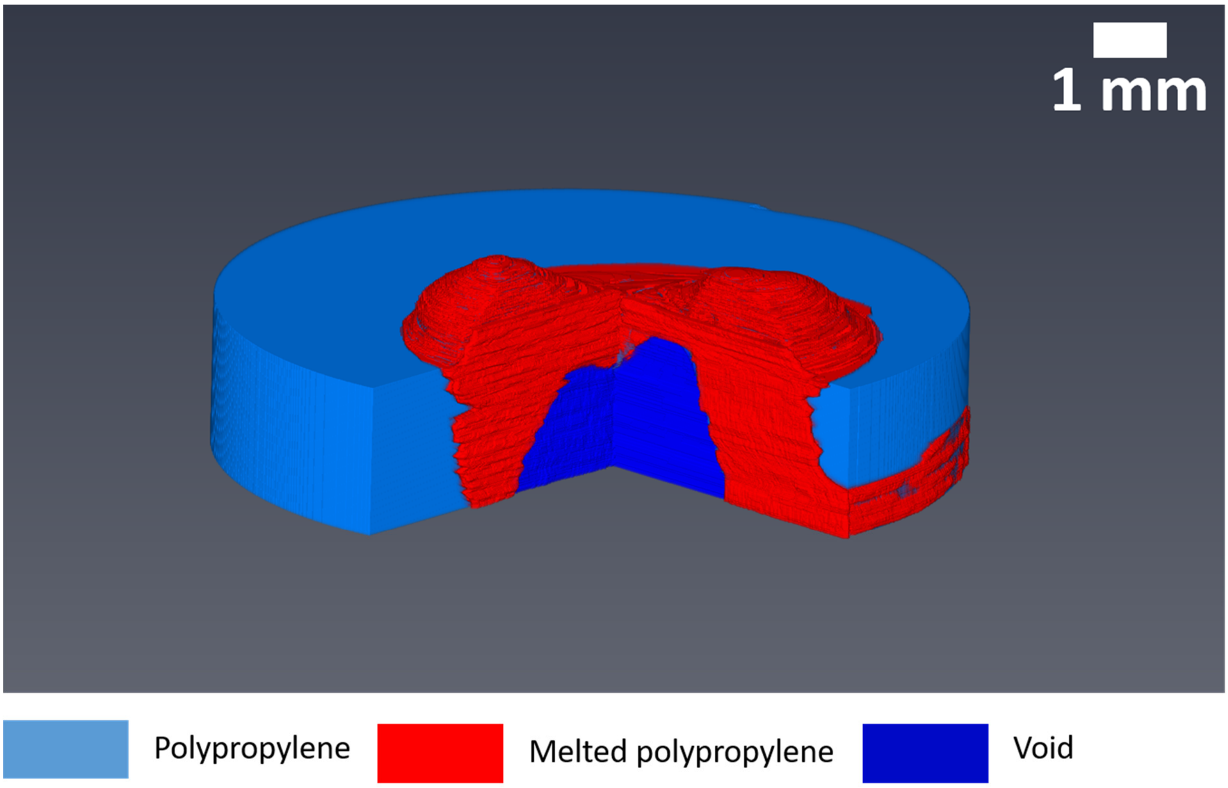

Figure S1. The 3D segmented image of the polypropylene sample at 58 s of laser irradiation partitioned into 3 regions. Light blue: polypropylene, red: melted polypropylene, and dark blue: void.

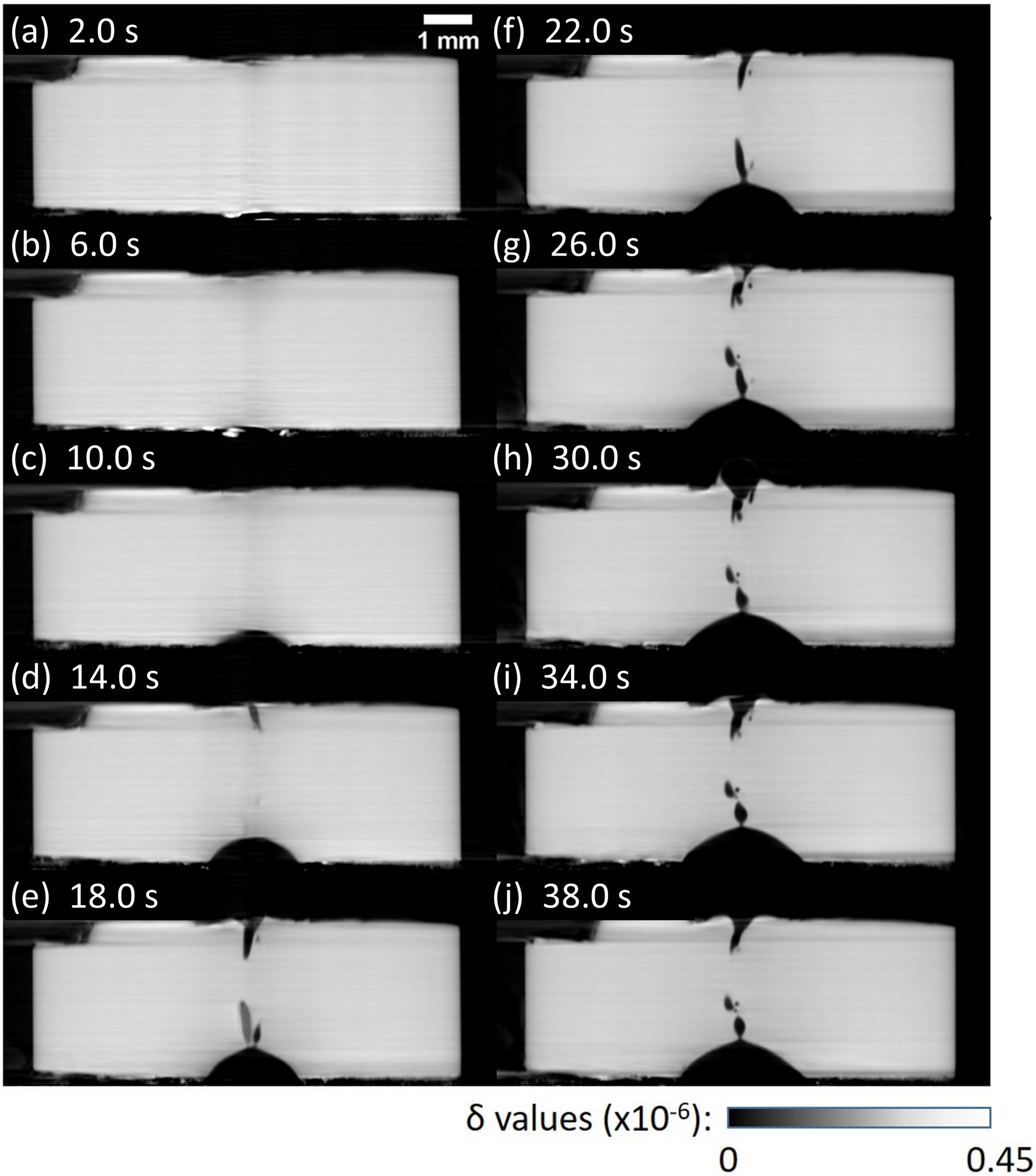

Figure S2. Sagittal section views of 4D phase tomograms obtained for the PMMA sample at different laser irradiation times in CW mode: (a) 2 s, (b) 6 s, (c) 10 s, (d) 14 s, (e) 18 s, (f) 22 s, (g) 26 s, (h) 30 s, (i) 34 s, and (j) 38 s. The images suggest strong back-reflection of infrared laser radiation from the supporting Al rod.

Movie S3. A movie of sagittal section views of 4D phase tomograms obtained for the polypropylene during laser irradiation in CW mode.

Movie S4. A movie of sagittal section views of 4D phase tomograms obtained for the PMMA sample during laser irradiation in pulse mode.

Movie S5. A movie of sagittal section views of 4D phase tomograms obtained for the PC sample during laser irradiation in CW mode.
